# Energy-Efficient Hydraulic System for Hexapod Robot Based on Two-Level Pressure System for Oil Supply

**DOI:** 10.3390/biomimetics10030151

**Published:** 2025-03-01

**Authors:** Junkui Dong, Bo Jin, Ziqi Liu, Lei Chen

**Affiliations:** 1 School of Mechanical Engineering, Zhejiang Sci-Tech University, Hangzhou 310018, China; 2 State Key Laboratory of Fluid Power and Mechatronic Systems, Zhejiang University, Hangzhou 310027, China; bjin@zju.edu.cn (B.J.); 12025063@zju.edu.cn (L.C.); 3 Ningbo Innovation Center, Zhejiang University, Ningbo 315100, China; 4 College of Mechanical and Electrical Engineering, China Jiliang University, Hangzhou 310018, China; zqliu@cjlu.edu.cn

**Keywords:** hydraulic system, hexapod robot, energy saving, multiple-pressure supply

## Abstract

This article proposes a two-level pressure system (TPS) inspired by mammalian energy supply mechanisms to enhance the energy efficiency of hydraulic hexapod robots (HHRs), In contrast to traditional one-level pressure systems (OPSs), the TPS contains both high-pressure and low-pressure oil supplies, which can switch the oil supply pressure according to the actuator load to reduce throttling loss and improve energy efficiency. Additionally, the TPS adopts a separate-meter-in and separate-meter-out (SMISMO) method to manage flow and pressure switching for the actuators. This article also analyzes the energy transfer process of an HHR and establishes kinematic and hydraulic system models. The energy-saving and control performance of the TPS is verified through simulations and experiments. The results show that compared to the OPS, the TPS achieves a 28.8% reduction in energy consumption while imposing higher demands on control performance.

## 1. Introduction

Multi-legged robots offer superior flexibility and mobility in complex uneven-terrain environments inaccessible to wheeled or tracked vehicles [1]. Compared to quadruped robots, hexapod robots have a higher stability and load capacity, although at the cost of certain aspects of their dynamic performance [2]. Hydraulic hexapod robots (HHRs) exhibit superior force output capabilities, power-to-weight ratios, and control robustness through hydraulic actuation. These advantages make HHRs particularly suitable for executing heavy-load tasks and performing critical missions in disaster response scenarios. However, hydraulic actuation also introduces increased complexity and control challenges, lower energy efficiency, and higher energy consumption. Consequently, energy-saving strategies for hydraulic robots have become a critical area of research.

Many advanced hydraulic legged robots have been successfully researched and developed, such as Boston Dynamics’ Big Dog [3]; IIT’s HyQ [4], HyQ2Max [5], and miniHyQ [6]; Shandong University’s SCalf [7,8]; Chiba University’s COMET [9,10]; NTUA’s HexaTerra [11]; and SJTU’s Baby Elephant [12], JINPOONG [13], LSHDSL-robot [14], and MBBOT [15]. While hydraulic legged robots exhibit superior force output and power-to-weight ratios, their energy conversion efficiency remains markedly inferior to their biological counterparts of comparable masses under most working conditions [16].

At present, three primary methods are employed to reduce the energy consumption of hydraulic legged robots: (1) the optimization of gait and foot trajectories; (2) the optimization of their mechanical structure; and (3) the optimization of their hydraulic actuators and hydraulic system architecture.

Optimization of gait and foot trajectories: Yang et al. [17] developed a model incorporating both mechanical power and heat rate for the SCalf-III. By optimizing the foot trajectory with the objective of energy efficiency, they achieved a 7.55% reduction in the robot’s energy consumption. Hua et al. [18] proposed an energy-efficient gait based on the segmented spline interpolation curve, enabling the SCalf-II to maintain low energy consumption on unknown terrain. Tani et al. [19] developed a novel approach to the trajectory design of hydraulic legged robots by incorporating the properties of limited-power hydraulic pumps. This method effectively improves energy utilization while ensuring better power matching. Optimization of the mechanical structure: Zhai et al. [20] proposed an archive-based micro genetic algorithm (AMGA) to optimize mechanical structure and gait parameters, resulting in a 40% reduction in energy consumption compared to the original structure. Liu et al. [21] presented a switchable parallel elastic actuator design for the monopedal switchable parallel elastic actuator robot (SPEAR), which adjusts the stiffness of the parallel elastic actuators to enhance energy efficiency without compromising mobility. Yin et al. [22] designed an origami-based decoupling clutch for energy-efficient legged robots, which switches the connection between the parallel spring and the robotic leg without requiring additional control effort. This decoupling clutch reduces the required knee flexion torque and energy consumption by 37.6% and 31%, respectively. Mazumdar et al. [23,24] developed a novel parallel spring mechanism to enhance energy efficiency in bipedal robots. Optimization of the hydraulic actuators and hydraulic system architecture: Fan et al. [25,26] proposed an unpowered hydraulic auxiliary system to enhance the loading capability and energy efficiency of legged robots. This system provides continuous support force to assist the knee joint actuator in bearing the load without consuming additional energy, resulting in a 16.9% improvement in energy efficiency during walking for a bipedal hydraulic-assisted electric leg prototype (HyELeg). Both Xue et al. [27] and Hua et al. [28] have developed multi-pressure hydraulic systems for hydraulic quadruped robots to reduce energy consumption. Xue et al. [29] proposed a novel variable-effective-area cylinder that reduces overall machine energy consumption by reducing the throttling loss and allowing energy recovery. Hua et al. [30] developed a hydraulically compliant servo actuator (HPCA) to enhance energy efficiency by utilizing the characteristics of the pressure-transition dynamics of an asymmetric hydraulic cylinder with a symmetrical valve configuration.

To improve the low energy efficiency of hydraulic legged robots, this article designs a hydraulic hexapod robot equipped with a two-level pressure system (TPS) with separate-meter-in and separate-meter-out (SMISMO) configurations. Previous studies have investigated the control performance of robotic joints by employing the TPS [31]. Compared to the traditional one-level pressure system (OPS) commonly employed in hydraulic robots, the TPS comprises high-pressure and low-pressure oil sources, which supply the actuator with the required pressure, which is based on load requirements. Both simulation and experimental results demonstrate that the TPS exhibits superior energy efficiency and an adequate control performance compared to the OPS, confirming that the TPS can replace the OPS to achieve stable and energy-efficient robotic motion.

In summary, the main contributions of this article are as follows: (1) the proposal of the TPS, inspired by mammalian energy supply mechanisms, to improve energy efficiency in hydraulic robots and (2) the verification of the TPS’s energy-saving and control performance, which demonstrates that the TPS can replace the OPS to enable stable and energy-efficient robotic motion.

The remainder of this article is structured as follows: Section 2 analyzes the energy transfer process and energy loss of the robot. Section 3 introduces an overview of the HHR, including its mechanical structure, hydraulic system architecture, and control system configuration. Section 4 establishes the kinematics model and hydraulic model of the HHR. Section 5 presents the energy-saving and control performance of both hydraulic systems through simulation and experimental results. Section 6 concludes this article.

## 2. Energy Consumption Analysis

**Figure 1:**
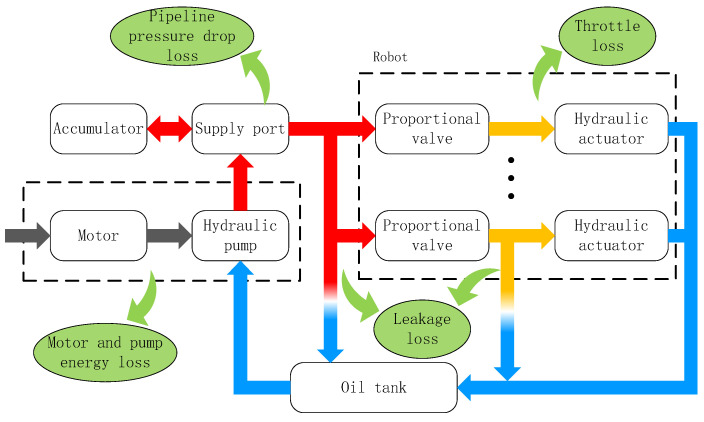
The energy transfer processes of HHRs.

In hydraulic legged robots, the hydraulic pump initially transforms mechanical energy into hydraulic fluid energy, which is subsequently converted back to mechanical energy through actuator systems. As illustrated in Figure 1, these energy conversion processes result in various forms of power dissipation. Prior to implementing energy efficiency optimization, a critical analysis is required to identify the primary loss mechanisms and assess their potential for energy saving. For analytical clarity, these losses are quantified in terms of power rather than cumulative energy. The total energy loss can be calculated by integrating the power loss over the duration of operation.

### 2.1. Motor and Pump Energy Loss

The sample manual provides the efficiency of the motor and pump, as well as the leakage coefficients of the hydraulic components. Therefore, the motor and pump energy loss can be calculated as follows:

(1) $$P_{MP}=\left(1-\eta_{t}\right)P_{s}Q_{s}$$

where $\eta_{t}$ is the comprehensive efficiency of the motor and the pump and $P_{s}$ and $Q_{s}$ are the system’s pressure and flow.

### 2.2. Pipeline Pressure Drop Loss

In the valve control system, the oil flowing through the pipeline induces a pressure drop, resulting in energy loss. Assuming the pipeline specifications are the same, the pipeline pressure drop loss can be expressed as follows:

(2) $$P_{PL}=\Delta P_{d}Q_{s}=\lambda \frac{l_{pi}}{d}\frac{\rho v^{2}}{2}Q_{p}=\lambda \rho \frac{8}{\pi^{2}}\frac{{Q_{p}}^{3}}{d^{5}}l_{pi}$$

where $\Delta P_{d}$ is the pressure drop; λ is the fluid resistance coefficient; $l_{pi}$ and *d* are the length and diameter of the pipeline, respectively; $Q_{p}$ and *v* are the flow rate and velocity of the oil through the pipeline; and ρ is the oil’s density.

### 2.3. Leakage Loss

Proportional valves exhibit inevitable internal leakage caused by mechanical clearance. For the ith valve, the leakage flow $\left(Q_{l,i}\right)$ demonstrates proportionality to both the valve leakage coefficient $\left(K_{lvi}\right)$ and the pressure differential $\left(\Delta P=P_{s}\right)$ across the valve. The total leakage loss is calculated as follows:

(3) $$P_{LL}=\sum_{i=1}^{N}\Delta PQ_{l,i}=\sum_{i=1}^{N}K_{lvi}{P_{s}}^{2}$$

### 2.4. Throttle Loss

In valve control systems, the spool displacement of proportional valves is regulated to supply the actuator with the required flow rate. However, a small displacement of the valve spool typically results in a pressure drop, leading to throttle loss. The throttle loss can be quantified based on the relationship between the required mechanical power for robotic motion and the hydraulic power transmitted through the valve. The throttle loss can be expressed as follows:

(4) $$P_{TL}=\sum_{i=1}^{M}\left(P_{s}Q_{a,i}-F_{a,i}{\dot{x}}_{a,i}\right)$$

where $Q_{a,i}$ representw the flow rate supplied to the ith actuator via the valve and $F_{a,i}$ and ${\dot{x}}_{a,i}$ represent the corresponding actuator’s load force and piston velocity, respectively.

### 2.5. Total Power Loss

The main types of power loss seen during the energy transfer process have been introduced. Based on Formulas (1)–(4), the total power loss can be expressed as follows:

(5) $$\begin{array}{cc} P_{total} & =P_{MP}+P_{PL}+P_{LL}+P_{TL}\\ & =\left(1-\eta_{t}\right)P_{s}Q_{s}+\lambda \rho \frac{8}{\pi^{2}}\frac{{Q_{s}}^{3}}{d^{5}}l_{pi}+\sum_{i=1}^{N}K_{lvi}{P_{s}}^{2}+\sum_{i=1}^{M}\left(P_{s}Q_{a,i}-F_{a,i}{\dot{x}}_{a,i}\right) \end{array}$$

The partial parameters in the formula correspond to fundamental hardware properties. The simplified expression, after incorporating these parameters, can be expressed as

(6) $$P_{total}=k_{MP}P_{s}Q_{s}+k_{PL}{Q_{s}}^{3}+k_{LL}{P_{s}}^{2}+\sum_{i=1}^{M}\left(P_{s}Q_{a,i}-P_{motion}\right)$$

where $k_{MP}$ represents the motor and pump loss coefficient; $k_{PL}$ represents the pipeline pressure drop loss coefficient; $k_{LL}$ represents the leakage loss coefficient; and $P_{motion}$ represents the power required for hydraulic actuator movement.

The formula indicates that enhancing the performance of the robot’s drive equipment, control valves, and actuators can reduce unnecessary losses. However, most research cannot balance energy efficiency and cost performance. Furthermore, achieving high integration and performance in the design of hydraulic components remains a significant challenge. Therefore, most research focuses on the impact of mechanical structures, hydraulic system design, and motion planning on the energy consumption of robots, focusing on the reduction of the flow rate supplied to the actuator and the power required for the robot to move.

This article focuses on system pressure and reduces throttling losses by optimizing the hydraulic system structure to match the system pressure to the actuator’s required pressure. It also proposes a TPS with SMISMO configurations, which can enhance robot energy efficiency by reducing throttling loss.

Compared to traditional hydraulic systems, this system can provide high-pressure or low-pressure oil sources to the hydraulic actuators based on the force conditions of the robot leg, which improves the robot’s pressure matching under different conditions. The specific robot model and hydraulic system model used will be introduced in the following sections.

## 3. Hydraulic Hexapod Robot Overview

### 3.1. Mechanical Structure

**Figure 2:**
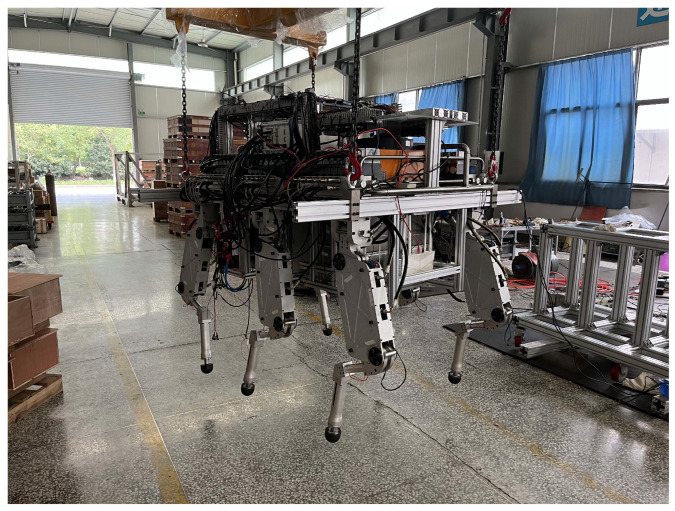
The hydraulic hexapod robot ZJUHEX01.

Animals have evolved structural adaptations to their environment through extended evolutionary processes. Hexapod robots can be primarily classified as insect-inspired and mammal-inspired types of robots based on their leg configuration and structural layout. Insect-inspired hexapods typically feature laterally distributed legs with a lower center of mass, demonstrating superior static stability and terrain adaptability at the expense of locomotion speed. In contrast, mammal-inspired hexapods possess more robust, vertically aligned legs beneath the body with a higher center of mass, exhibiting superior advantages in movement velocity, obstacle-crossing capability, and load capacity. The HHR shown in Figure 2 emulates the biomechanical architecture of mammals and comprises a central body and six identical legs, each with a three-degree-of-freedom design. The size of this HHR is about 1.65 m (L) × 1 m (W) × 1.6 m (H) at its standard standing position, and its total mass is about 800 kg, with an extra load capability of about 300 kg.

### 3.2. Hydraulic System Structure

**Figure 3:**
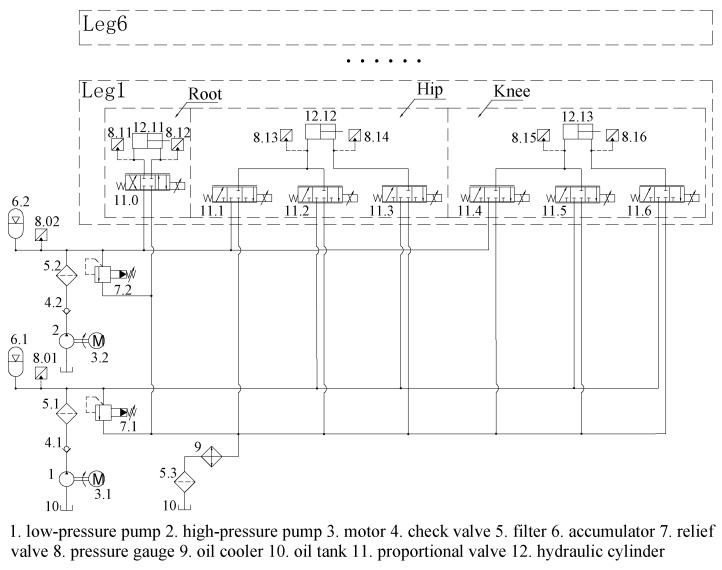
Schematic of the hydraulic system with two-level pressure.

With hydraulic legged robots, much attention has been paid to imitating the structure and movement patterns of humans and animals. However, little attention is paid to imitating their power supply method. Therefore, most of the robot’s energy optimization is focused on its structure and motion trajectory, and only a small part is focused on its hydraulic system. Furthermore, now most hydraulic legged robots have only one oil source and multiple actuators, which causes a large amount of throttling loss. This article proposes a biomimetic hydraulic system that enhances robotic energy efficiency by emulating mammalian metabolic energy strategies. In biological systems, aerobic metabolism maintains a continuous low-power supply, while anaerobic metabolism delivers instantaneous high-power output. Inspired by this metabolic principle, this article develops the TPS as illustrated in Figure 3. This system employs a two-level pressure architecture, where a high-pressure source emulates anaerobic metabolism to power high-load hydraulic actuators, while a low-pressure source mimics aerobic metabolism to enable the energy-efficient operation of low-load actuators. To achieve coordinated pressure switching and motion control, the TPS uses an SMISMO configuration.

As shown in Figure 3, the TPS consists of two pumps that provide high pressure and low pressure for the actuators’ various output force requirements. Each robot leg comprises a root joint, hip joint, and knee joint. Considering the relatively small displacement and flow requirements of the root actuator during straight gaits, the root joints are supplied with high-pressure oil, while the knee and hip joints are supplied with both high-pressure and low-pressure oil. As a result, the root joints adopt a traditional valve-controlled cylinder system, whereas the knee and hip joints adopt a SMISMO system.

In the support phase, when the leg needs to support the body, the actuators in the leg are supplied with high-pressure oil to meet locomotion requirements. When the leg is in the swing phase and only needs to overcome gravity and dynamic force, the actuators in the leg are supplied with low-pressure oil to reduce throttling loss and improve energy efficiency.

### 3.3. Control System Structure

**Figure 4:**
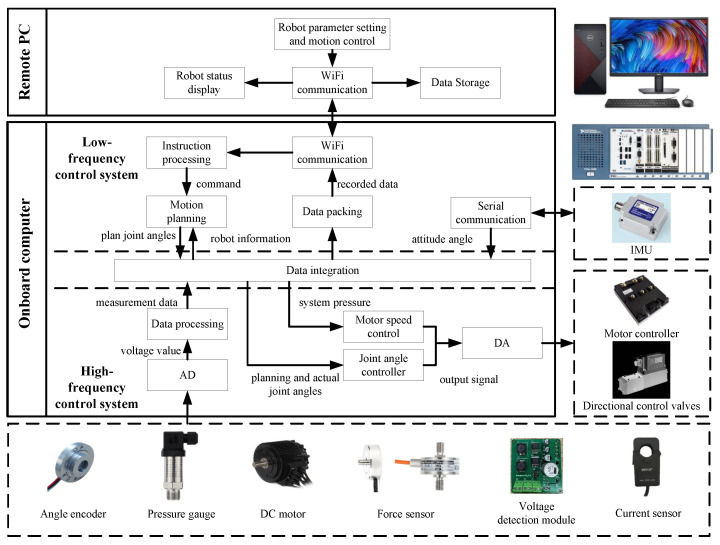
The entire measurement and control system of the HHR.

To achieve the HHR’s independent movement and remote control functions, this article proposes a high–low frequency composite hierarchical control system, which meets the robot’s control requirements of having multiple inputs and outputs, high real-time performance, and complex control computations. The entire measurement and control system is shown in Figure 4.

The system comprises a remote PC, an onboard computer, an inertial measurement unit (IMU), angular encoders, pressure gauges, force sensors, etc. The joint angles are measured by embedded encoders, while hydraulic pressure and robot attitude are measured through pressure sensors and IMUs. Foot force transducers detect ground reaction forces and determine leg contact states. The primary sensors employed in the control system are summarized in Table 1.

As the upper layer, the remote PC carries out the human–computer interaction function and communicates with the onboard computer via Wi-Fi. As the lower layer, the NI-PXI platform carries out the robot’s signal sampling and output, motion planning, gait and trajectory planning, and control algorithm application.

This article proposes this high–low frequency composite system to balance control performance, the system’s real-time performance, and computer performance. The different robot control tasks are divided into a low-frequency control system parts and a high-frequency control system parts. The system frequency of the low-frequency control system is 200 Hz, and this part completes complex robot motion control calculations and remote PC communication, with low real-time requirements. The system frequency of the high-frequency control system is 2000 Hz, and this part completes sensor data acquisition, joint angle control, and motor control.

## 4. System Modeling and Analysis

### 4.1. Kinematic Modeling

**Figure 5:**
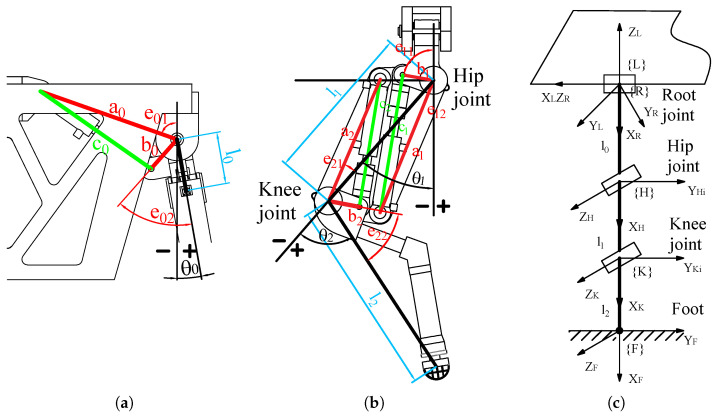
The structure and coordinate frames of the HHR’s leg. (**a**) The structure of the root joint; (**b**) the structure of the hip and knee joint; (**c**) the leg coordinate framework.

The six legs of the HHR have the same structure, differing only in their mounting orientation and position on the robot. Take the front left leg, for example, which we will use to explain the structure of the leg and the installation position of the hydraulic cylinders, as shown in Figure 5a,b. In that figure, $\theta_{0}$, $\theta_{1}$, and $\theta_{2}$ are the joint angle of the root, hip, and knee, respectively. $l_{1}$, $l_{2}$, and $l_{3}$ are the root length, thigh length, and calf length, respectively. $a_{*}$, $b_{*}$, and $e_{*}$ are the position parameters of the installed hydraulic cylinders.

The relationship between the hydraulic cylinders and joint angles can be written as follows:

(7) $$\left\{\begin{array}{c} c_{0}=\sqrt{a_{0}^{2}+b_{0}^{2}+2a_{0}b_{0}\cos\left(\theta_{0}-e_{01}-e_{02}\right)}\\ c_{1}=\sqrt{a_{1}^{2}+b_{1}^{2}+2a_{1}b_{1}\cos\left(\theta_{1}-e_{11}+e_{12}\right)}\\ c_{2}=\sqrt{a_{2}^{2}+b_{2}^{2}+2a_{2}b_{2}\cos\left(\theta_{2}-e_{21}+e_{22}\right)} \end{array}\right.$$

(8) $$\left\{\begin{array}{c} l_{c0}=-\frac{a_{0}b_{0}\sin\left(\theta_{0}-e_{01}-e_{02}\right)}{c_{0}}\\ l_{c1}=-\frac{a_{1}b_{1}\sin\left(\theta_{1}-e_{11}+e_{12}\right)}{c_{1}}\\ l_{c2}=\frac{a_{2}b_{2}\sin\left(\theta_{2}-e_{21}+e_{22}\right)}{c_{2}} \end{array}\right.$$

where $c_{0}$, $c_{1}$, and $c_{2}$ are the length of the root, hip, and knee joint’s hydraulic cylinders. $l_{c0}$, $l_{c1}$, and $l_{c2}$ are the lever arm length of the hydraulic cylinders’ output force.

We can simplify the leg structure and establish the coordinate system shown in Figure 5c. The coordinate system {L} is the leg coordinate system. The coordinate systems {R}, {H}, {K}, and {F} are the root joint coordinate system, the hip joint coordinate system, the knee joint coordinate system, and the foot coordinate system, respectively.

The transformation matrix from the foot coordinate system {F} to the leg coordinate system {L} can be written as follows:

(9) TFL=R(θ)P(θ)01

(10) $$R(\theta )\;=\left[\begin{array}{ccc} -\sin(\theta_{1}+\theta_{2}) & -\cos(\theta_{1}+\theta_{2}) & 0\\ \cos\left(\theta_{1}+\theta_{2}\right)\sin\theta_{0} & -\sin\left(\theta_{1}+\theta_{2}\right)\sin\theta_{0} & \cos\theta_{0}\\ -\cos\left(\theta_{1}+\theta_{2}\right)\cos\theta_{0} & \sin\left(\theta_{1}+\theta_{2}\right)\cos\theta_{0} & \sin\theta_{0} \end{array}\right]$$

(11) $$P(\theta )\;=\left[\begin{array}{c} -l_{1}\sin\theta_{1}-l_{2}\sin\left(\theta_{1}+\theta_{2}\right)\\ \left[l_{0}+l_{1}\cos\theta_{1}-l_{2}\cos\left(\theta_{1}+\theta_{2}\right)\right]\sin\theta_{0}\\ -\left[l_{0}+l_{1}\cos\theta_{1}-l_{2}\cos\left(\theta_{1}+\theta_{2}\right)\right]\cos\theta_{0} \end{array}\right]=\left[\begin{array}{c} P_{x}\\ P_{y}\\ P_{z} \end{array}\right]$$

where P(θ) is the foot’s position in the leg coordinate system.

Based on formula (11), the joint angles are formulated by inverse kinematics calculations, as follows:

(12) $$\theta_{0}=-\arctan\left(\frac{P_{y}}{P_{z}}\right)$$

(13) $$\theta_{1}=\arctan\left(\frac{P_{x}l_{1}\cos\theta_{0}+P_{x}l_{2}\cos\theta_{0}\cos\theta_{2}-\left(P_{z}+l_{0}\cos\theta_{0}\right)l_{2}\sin\theta_{2}}{l_{1}\left(P_{z}+l_{0}\cos\theta_{0}\right)+l_{2}\cos\theta_{2}\left(P_{z}+l_{0}\cos\theta_{0}\right)+l_{2}P_{x}\cos\theta_{0}\sin\theta_{2}}\right)$$

(14) $$\theta_{2}=\arccos\left(\frac{l^{2}+{P_{x}}^{2}-{l_{1}}^{2}-{l_{2}}^{2}}{2l_{1}l_{2}}\right)$$

where *l* is the intermediate variable for length transformation, $l=\sqrt{{P_{y}}^{2}+{P_{z}}^{2}}-l_{0}$.

### 4.2. Hydraulic System Modeling

#### 4.2.1. SMISMO System Modeling

**Figure 6:**
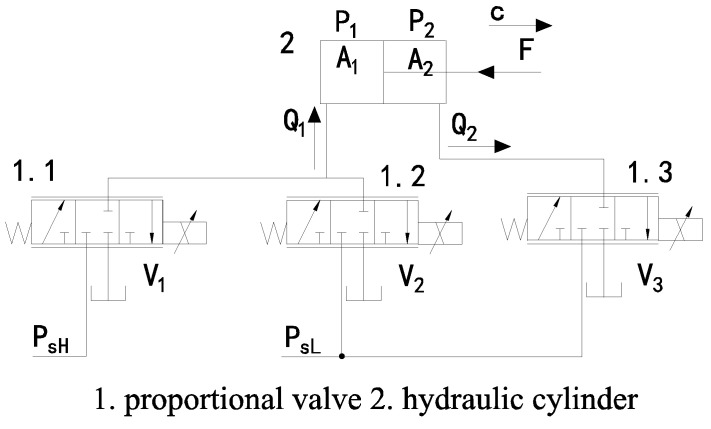
Schematic of the simplified SMISMO system.

In contrast to conventional valve-controlled cylinder configurations, the SMISMO system employs multiple valves for the independent regulation of meter-in and meter-out orifices, enhancing both control flexibility and energy efficiency. A simplified hydraulic system for the hip joint (Figure 6) is presented to elucidate the operational principles of this SMISMO configuration. $Q_{1}$ and $Q_{2}$ are the flow entering the cylinder chamber without the rod and the flow leaving the cylinder chamber with the rod. Valve $V_{1}$ provides high-pressure oil, while the valves $V_{2}$ and $V_{3}$ offer low-pressure oil.

Assuming the valves are ideal, and ignoring the leakage of the hydraulic system, the flow rates of the hydraulic cylinder with and without the rod can be written as follows:

(15) $$\left\{\begin{array}{c} Q_{1}=Q_{v_{1}}+Q_{v_{2}}\\ Q_{2}=Q_{v_{3}} \end{array}\right..$$

(16) $$\left\{\begin{array}{c} Q_{v_{1}}=\left\{\begin{array}{c} k_{q}x_{v_{1}}\sqrt{|P_{sH}-P_{1}|}\cdot sign\left(P_{sH}-P_{1}\right)\\ k_{q}x_{v_{1}}\sqrt{|P_{1}-P_{t}|}\cdot sign\left(P_{1}-P_{t}\right) \end{array}\right.\begin{array}{c} ,x_{v_{1}}\geq 0\\ ,x_{v_{1}}<0 \end{array}\\ Q_{v_{2}}=\left\{\begin{array}{c} k_{q}x_{v_{2}}\sqrt{|P_{sL}-P_{1}|}\cdot sign\left(P_{sL}-P_{1}\right)\\ k_{q}x_{v_{2}}\sqrt{|P_{1}-P_{t}|}\cdot sign\left(P_{1}-P_{t}\right) \end{array}\right.\begin{array}{c} ,x_{v_{2}}\geq 0\\ ,x_{v_{2}}<0 \end{array}\\ Q_{v_{3}}=\left\{\begin{array}{c} -k_{q}x_{v_{3}}\sqrt{|P_{sL}-P_{2}|}\cdot sign\left(P_{sL}-P_{2}\right)\\ -k_{q}x_{v_{3}}\sqrt{|P_{2}-P_{t}|}\cdot sign\left(P_{2}-P_{t}\right) \end{array}\right.\begin{array}{c} ,x_{v_{3}}\geq 0\\ ,x_{v_{3}}<0 \end{array} \end{array}\right.$$

where $Q_{v_{1}}$, $Q_{v_{2}}$, a nd $Q_{v_{3}}$ are the flow rate of the three proportional directional control valves $V_{1}$, $V_{2}$, and $V_{3}$; $P_{1}$ and $P_{2}$ are the hydraulic cylinder’s pressure without and with the rod; and $P_{sH}$, $P_{sL}$, and $P_{t}$ are the pressure of the high-pressure oil supply, the low-pressure oil supply, and the oil tank. $x_{v_{1}}$, $x_{v_{2}}$, and $x_{v_{3}}$ are the spool positions of the valves $V_{1}$, $V_{2}$, and $V_{3}$.

The dynamic frequency of the valve significantly exceeds that of the robot’s mechanical system. Therefore, the dynamic characteristics of valves are typically modeled as a second-order system.

(17) $$\frac{x_{v}}{u_{iv}}=\frac{{\omega_{v}}^{2}}{s^{2}+2\zeta \omega_{v}s+{\omega_{v}}^{2}}$$

where $u_{iv}$ is the voltage of the control valve, $\omega_{v}$ is the natural frequency of the valve, and ζ is the damping coefficient.

Ignoring leakage and based on the flow continuity equation, the pressure dynamic equation of the hydraulic cylinder can be written as

(18) $$\frac{V_{c1}}{\beta_{e}}{\dot{P}}_{1}=Q_{1}-A_{1}\dot{c}$$

(19) $$\frac{V_{c2}}{\beta_{e}}{\dot{P}}_{2}=-Q_{2}+A_{2}\dot{c}$$

where $V_{c1}=V_{pl1}+A_{1}c$ and $V_{c2}=V_{pl2}+A_{2}\left(L-c\right)$; $V_{c1}$ and $V_{c2}$ are the hydraulic cylinder volume without and with the rod; $V_{pl1}$ and $V_{pl2}$ are the volume of the cavity between the valves and the hydraulic cylinder; $\beta_{e}$ is the bulk modulus of the hydraulic oil; $A_{1}$ and $A_{2}$ are the hydraulic cylinder areas without the rod and with the rod; and $\dot{c}$ is the piston velocity of the hydraulic cylinder.

The output force of the hydraulic cylinder can be written as follows:

(20) $$F_{cyl}=A_{1}P_{1}-A_{2}P_{2}-F_{cf}$$

where $F_{cf}$ is the hydraulic cylinder’s friction, which can be estimated based on the literature [17].

This article primarily investigates the energy consumption of the robot. Both the robot joints and hydraulic valves are controlled using PID controllers, with the control signals distributed as below.

When the robot leg is in its support phase,

(21) $$\left\{\begin{array}{c} u_{1v}=u_{ikPID}\\ u_{2v}=0\\ u_{3v}=-u_{ikPID} \end{array}\right.$$

When the robot leg is in its swing phase,

(22) $$\left\{\begin{array}{c} u_{1v}=0\\ u_{2v}=u_{ikPID}\\ u_{3v}=-u_{ikPID} \end{array}\right.$$

#### 4.2.2. Energy Modeling of the Hydraulic System

This article investigates the impact of system pressure and throttling loss on energy consumption. The hydraulic system’s pressure can be adjusted through variable-speed control of the motor and pump. To simplify the analysis, the effects of the hydraulic oil’s elastic volume expansion and the leakage of the hydraulic components are neglected, and the system pressures are constant. Based on the research in reference [32], the system pressures of TPSs and OPSs are determined by the peak pressure demand of the actuators during operational cycles, with a 3 MPa pressure margin. The total power of the hydraulic system can be expressed as

(23) $$\begin{array}{c} P_{sys}=P_{s}Q_{s}=P_{s}\sum_{i=1}^{M}Q_{a,i} \end{array}$$

where $Q_{s}$ is the hydraulic system’s flow and $Q_{a,i}$ is the flow rate delivered to the ith actuator through the valve, which can be estimated based on the actuator’s motion.

(24) $$Q_{a,i}=\left\{\begin{array}{c} \begin{array}{cc} A_{1}{\dot{c}}_{i} & {\dot{c}}_{i}>0\\ -A_{2}{\dot{c}}_{i} & {\dot{c}}_{i}<0 \end{array} \end{array}\right.$$

where $A_{1}$ and $A_{2}$ are the hydraulic cylinder areas without the rod and with the rod and $\dot{c}$ is the piston velocity of the hydraulic cylinder.

The motor input power can be estimated based on the hydraulic system’s power and the combined efficiency of the motor and pump.

(25) $$P_{motor}=\frac{P_{sys}}{\eta_{t}}$$

where $\eta_{t}$ is the comprehensive efficiency of the motor and the pump.

### 4.3. Trajectory Planning

**Figure 7:**
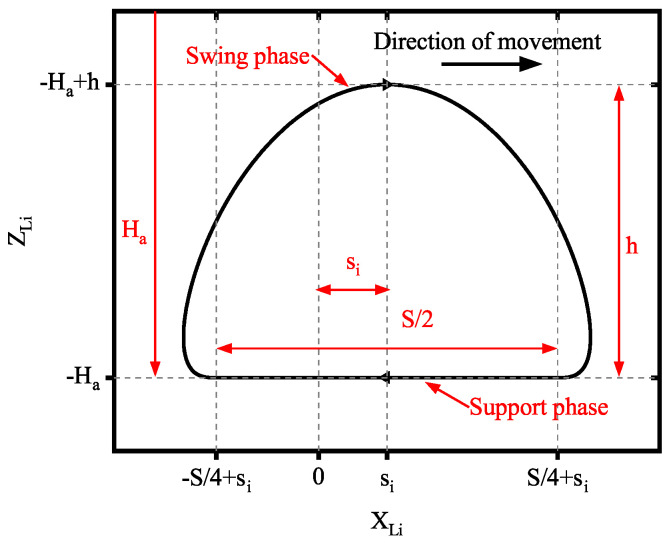
The foot trajectory curve.

The hexapod robot’s motion trajectory and duty factor significantly influence its energy consumption during locomotion. To better compare the energy consumption of the traditional OPS and the TPS, the robot uses a tripod gait with a 0.5 duty factor for constant-speed linear movement. The foot trajectory is described below, and its curve is shown in Figure 7.

The swing-phase foot trajectory ($0<t\leq \frac{T}{2}$) of the robot:

(26) $$\left\{\begin{array}{c} X_{Li}\left(t\right)=\left(a_{s0}+a_{s1}t+a_{s2}t^{2}+a_{s3}t^{3}+a_{s4}t^{4}+a_{s5}t^{5}+a_{s6}t^{6}\right)S+s_{i}\\ Y_{Li}\left(t\right)=0\\ Z_{Li}\left(t\right)=\left(b_{s0}+b_{s1}t+b_{s2}t^{2}+b_{s3}t^{3}+b_{s4}t^{4}+b_{s5}t^{5}+b_{s6}t^{6}\right)h-H_{a} \end{array}\right.$$

The stance-phase foot trajectory ($\frac{T}{2}<t\leq T$) of the robot:

(27) $$\left\{\begin{array}{c} X_{Li}\left(t\right)=\left(\frac{3}{4}-\frac{t}{T}\right)S+s_{i}\\ Y_{Li}\left(t\right)=0\\ Z_{Li}\left(t\right)=-H_{a} \end{array}\right.$$

where $X_{Li}\left(t\right)$, $Y_{Li}\left(t\right)$, and $Z_{Li}\left(t\right)$ are the foot trajectory in the leg coordinate system; *S*, $s_{i}$, *h*, $H_{a}$, and *T* are the step length, offset, step height, robot height, and the cycle of the gait, respectively; and $a_{si}$, $b_{si}$(i=0∼6) are the six-order coefficients of the polynomial designed using the constraints in Table 2.

## 5. Results and Discussion

### 5.1. Simulation

**Figure 8:**
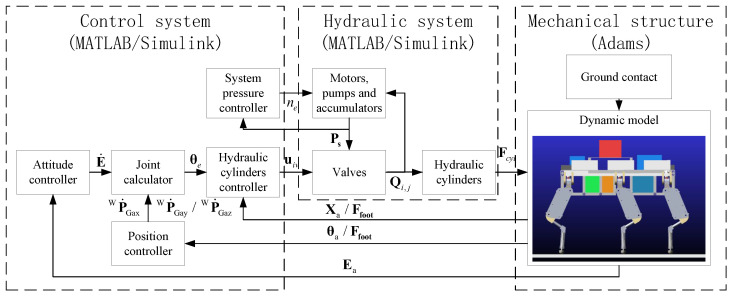
The virtual prototype of HHR.

The HHR’s co-simulation architecture includes the control system, hydraulic system, and mechanical model. The control system and hydraulic system were developed in MATLAB/Simulink, while the mechanical model was built in Adams. The schematic of this co-simulation architecture is provided in Figure 8.

The interface for the interaction between Adams and MATLAB/Simulink is encapsulated in an adams_sub module. This module transmits the output forces of the hydraulic system as inputs to Adams while returning the parameters of the robot simulation dynamics as output to MATLAB/Simulink. The hydraulic system parameters are shown in Table 3. In the mechanical model, the proper configuration of the ground contact model is crucial, as it directly influences the foot contact force and impacts the motion of the HHR. The relevant parameters are listed in Table 4.

**Figure 9:**
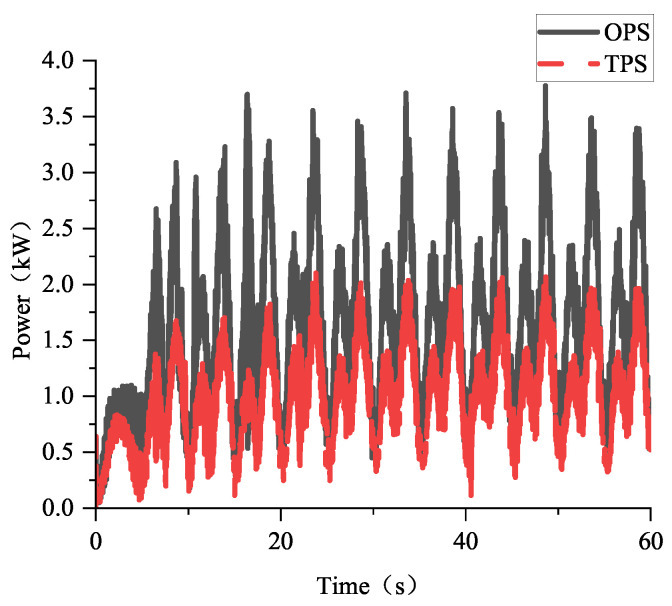
The comparison of the simulated power of the OPS and TPS.

**Figure 10:**
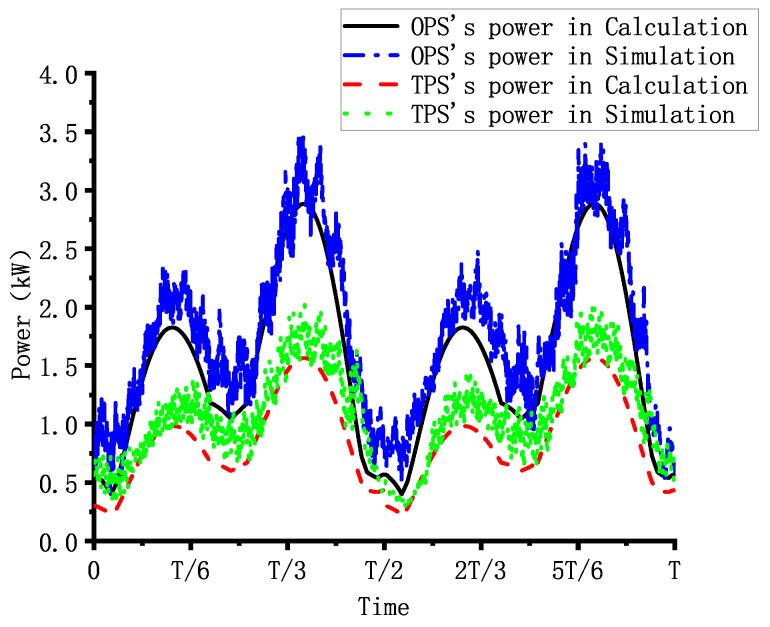
The power comparison of OPS and TPS in simulation and calculation during one cycle.

In the simulation, the high pressure and low pressure of the TPS are 16 MPa and 8 MPa, respectively, while the system pressure of the OPS is 16 MPa. The robot moves using a preplanned tripod gait at a speed of 0.05 m/s and with a cycle time of 10 s. The total simulation time is 60 s, with the first 20 s allocated for robot posture adjustment, followed by 40 s of constant-speed linear motion. Figure 9 shows the power consumption of the robot when it is equipped with the OPS or the TPS during the simulation. And Figure 10 presents the calculated, based on Formula (25), and simulated power of the robot, when equipped with the two different hydraulic systems, within a single gait cycle. Based on the simulation results, the average power of the robot during constant-speed linear motion can be calculated. The average power of the robot with the TPS is 1.079 kW, representing a 39.13% reduction compared to the 1.77 kW average power needed with the OPS. Additionally, the peak power of the robot is reduced by 41.3% with the TPS compared to the OPS.

**Figure 11:**
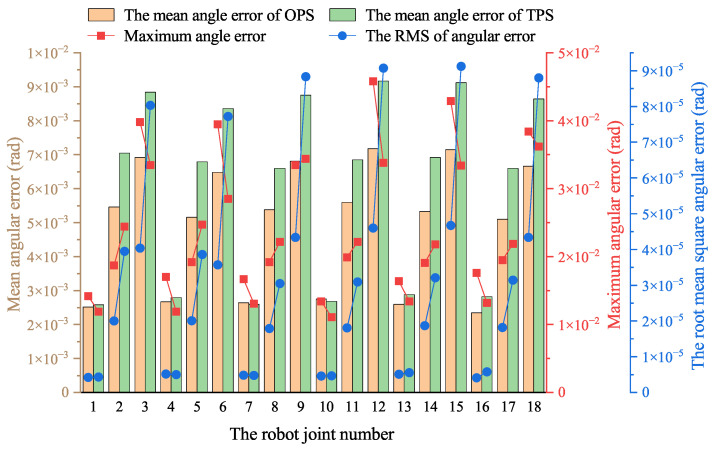
Mean angular error, maximum angular error, and root mean square of the angular error of robot joint angles equipped with OPS or TPS during simulation.

Compared to the OPS, the TPS offers high-pressure and low-pressure oil sources, enhancing energy efficiency by reducing throttling loss. However, using two-level pressure of the oil sources inevitably results in pressure fluctuations during valve switching, which can impact the robot’s control accuracy. From an energy-saving perspective, a slight reduction in control precision is acceptable to enhance the robot’s energy efficiency. The simulation results demonstrate that the TPS shows a superior energy-saving performance compared to the OPS, necessitating a comparison of the control performance of the two hydraulic systems. Figure 11 illustrates the mean angular error, maximum angular error, and root mean square (RMS) of the joint angle error of the robot when equipped with the OPS or TPS in simulation.

As shown in Figure 11, the mean joint angle errors in the TPS are generally higher than those in the OPS, while the maximum error varies. Considering potential contingencies in the simulation, these results indicate that the control performance of the two hydraulic systems is comparable and within an acceptable range. Additionally, the RMS of the angular error is greater in the TPS than in the OPS, suggesting that pressure surges during the switching between the two different-pressure oil sources affect the actuator’s control accuracy, leading to minor fluctuations in the joint angles.

By analyzing the power consumption and control performance of the two hydraulic systems, it is demonstrated that, compared to the traditional OPS, the TPS reduces throttling loss when the robot moves while maintaining acceptable control accuracy not compromising its motion performance. The TPS enhances the robot’s energy efficiency and reduces the power consumed during movement.

### 5.2. Experiment

**Figure 12:**
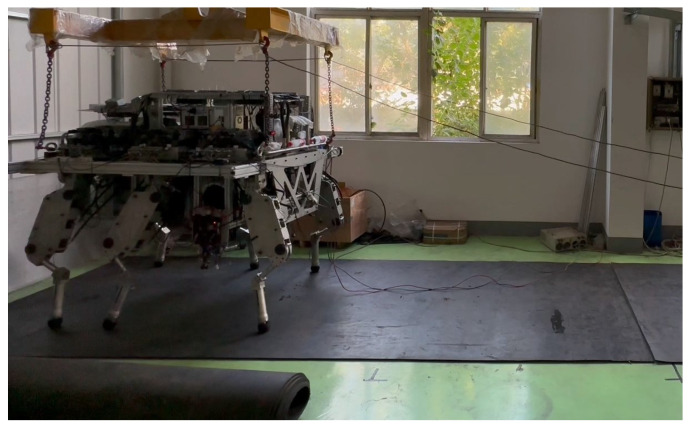
The experimental setup and environment.

To further validate the energy-saving performance of the proposed TPS, a physical experiment is conducted on an HHR equipped with the TPS. Figure 12 shows the experimental setup and environment of the HHR; the robot traversed from the left side to the right side of the test area. The structure of the HHR used in this experiment is outlined in Section 3. To reduce experimental costs, industrial-grade motors and proportional directional valves were employed, which may create certain performance limitations. Nevertheless, this also demonstrates that the TPS has relatively low hardware requirements, as it can achieve a satisfactory energy-saving performance even with hardware of limited capabilities. In the experiment, the robot’s OPS employed a traditional one-level pressure SMISMO hydraulic system, similar to the proposed TPS described in this article. The two hydraulic systems can be switched by changing the number of corresponding proportional valves. Additionally, the gait parameters used in the experiment are the same as those in the simulation.

Consistent with the simulation, the system pressure for the OPS used in the experiment was set to 16 MPa, while the TPS’s system pressures were set to 16 MPa and 8 MPa. To minimize the influence of structural and control factors on the robot’s motion, a preplanned tripod gait was employed to maintain constant-speed movement without adjustment phases.

**Figure 13:**
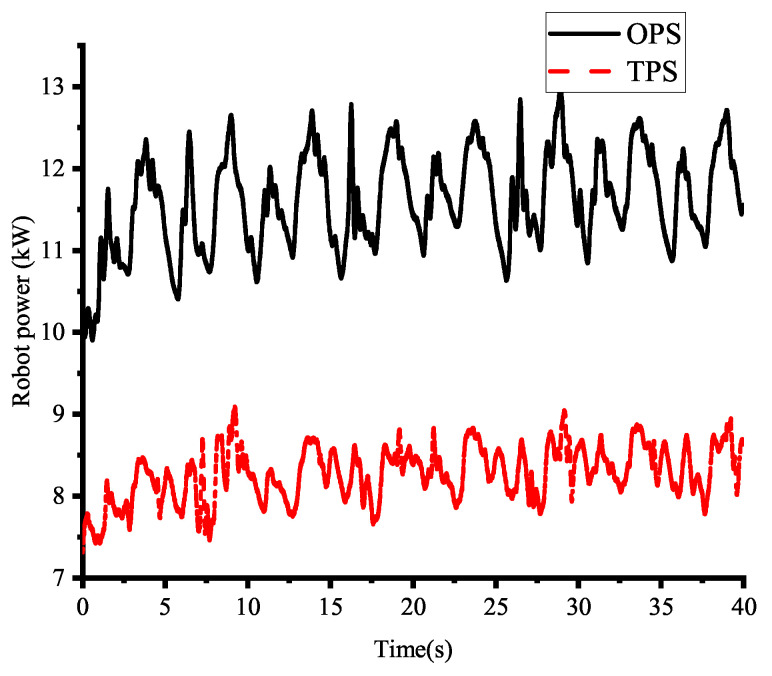
The power comparison of the OPS and TPS used in the experiment.

As illustrated in Figure 13, the robot’s motion power is calculated based on the motor’s input voltage and current, which include all power consumption losses described in Formula (5). Experimental data indicate that the average movement power consumption of the robot is 11.7 kW and 8.3 kW with the OPS and TPS, respectively. Compared to the OPS, the power consumption of the TPS is consistently lower throughout the motion, resulting in a 28.8% reduction in average power consumption.

The results indicate that the robot’s power consumption in the experiment is significantly higher than in the simulation, although the trend in power variation is the same. This discrepancy is primarily due to the simplifications made in the simulation, where leakage loss and pipeline pressure drops are neglected. Additionally, the industrial-grade proportional valves used in the experimental hydraulic system exhibit non-negligible leakage. Furthermore, damping orifices were used in the hydraulic system to help control the robot root joints, further exacerbating leakage loss. These factors collectively resulted in the experimental power consumption being substantially higher than in the simulation.

This article primarily focuses on the impact of the throttling loss on the robot’s energy consumption; therefore, the following assumptions are made: (1) throttling losses are negligible when the robot is stationary; (2) the combined efficiency of the motor and pump remains constant; and (3) the leakage loss and pipeline pressure drop loss are essentially unchanged. Based on these assumptions, the robot’s power consumption, including its throttling loss, can be estimated using the equation below.

(28) $$P_{et}=P_{ui}-P_{stop}$$

where $P_{et}$ is the estimated power used for the robot’s movement; $P_{ui}$ is the robot’s power, calculated based on the motor’s input voltage and current; and $P_{stop}$ is the power used by the robot while standing.

**Figure 14:**
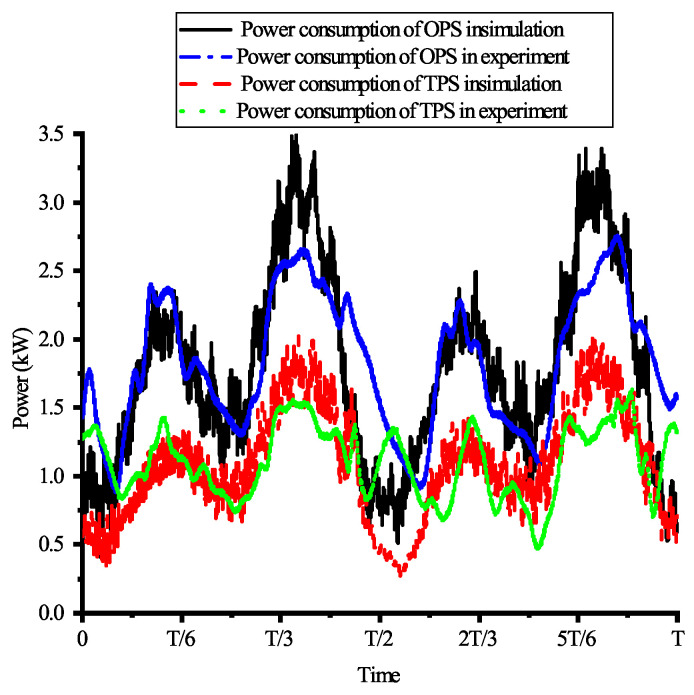
Comparison of the power consumption of the OPS and TPS during one cycle, as estimated from the simulation and experiment.

Using the method described above, the power consumption of the robot is estimated for both hydraulic systems. The TPS demonstrated a power consumption of 0.98 kW, representing a 42.7% reduction compared to the 1.71 kW of power consumed by the OPS. Figure 14 compares the estimated power consumption of both hydraulic systems in the experiment with their power consumption as simulated over one cycle. As illustrated in the figure, while there are discrepancies between the experimentally estimated power and the simulated power of the hydraulic systems, the overall trends remain consistent.

**Figure 15:**
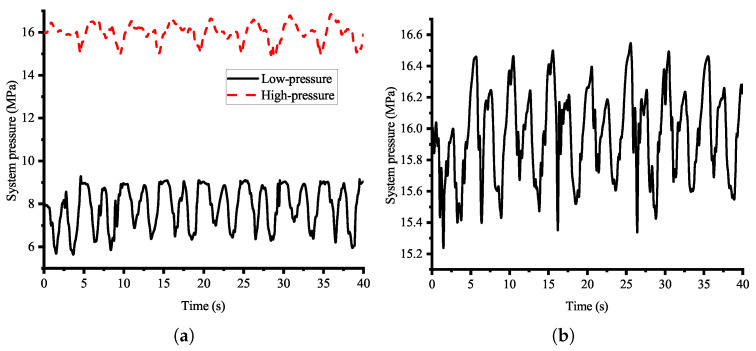
System pressure of both hydraulic systems during experiment. (**a**) TPS; (**b**) OPS.

**Figure 16:**
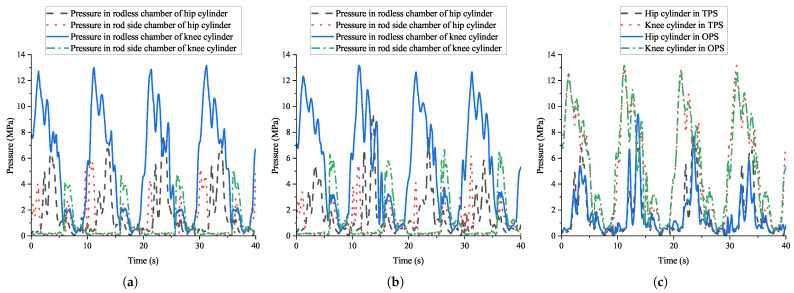
Cylinder chamber pressures of both systems during experiment. (**a**) TPS; (**b**) OPS; (**c**) comparison of rodless chamber pressure.

The experimental results indicate that the TPS significantly enhanced robotic energy efficiency by reducing throttling losses compared to the OPS. Given the TPS’s superior energy conservation characteristics, it is essential to evaluate both systems’ control performance to ensure they have comparable control capabilities. Figure 15 shows the system pressure variations of the TPS and OPS during the robot’s motion. The TPS’s average system pressures are 15.99 MPa and 7.89 MPa, respectively, while that of the OPS is 15.97 MPa. Both hydraulic systems exhibited similar high-pressure fluctuations. The effects of the two hydraulic systems on pressure variations in both chambers of the hydraulic cylinders during motion were analyzed. For clarity and reliability, the left middle leg with the highest load was selected as the representative case. Figure 16 shows the experimentally measured pressure in both chambers of the hip and knee joints’ cylinders. The results reveal that both chambers exhibit periodic pressure fluctuations with similar trends, and the period of these matches the 10 s gait cycle. This phase-synchronous fluctuation demonstrates comparable control characteristics between the two systems. These findings confirm that the pressure compatibility is enhanced when the cylinders are supplied by the low pressure of the TPS.

**Figure 17:**
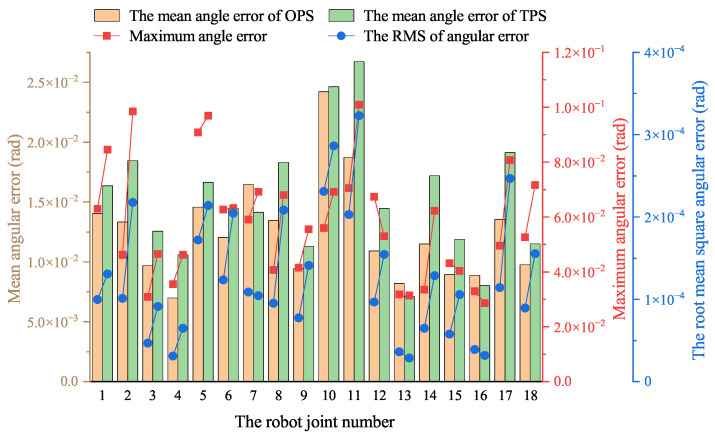
Mean angular error, maximum angular error, and root mean square of angular error of robot joint angles equipped with OPS and TPS during experiment.

Figure 17 illustrates the mean angular error, maximum angular error, and RMS of the robot’s joint angular error for both the OPS and TPS as measured in the experiment, which leads to similar conclusions as those made from the simulation. As shown in the figure, the mean joint angular errors and RMS of the TPS are generally higher than those of the OPS, while the maximum error varies. Considering this experiment’s complexity and potential accidentally dissimilar conditions, these results indicate that the control performance of the two hydraulic systems is comparable and within an acceptable range.

In conclusion, the TPS demonstrates superior energy efficiency while maintaining a comparable control performance to that of the OPS. Therefore, the TPS can replace the OPS to achieve stable, energy-efficient robotic motion.

## 6. Conclusions

This article proposes a hydraulic system with two pressure levels to reduce the large throttling loss in traditional single-pressure multi-actuator hydraulic systems and comprehensively analyzes the energy-saving principles and efficiency of this hydraulic system. An overview of the mechanical structure, hydraulic system structure, and control system structure of a robot equipped with the energy-efficient two-pressure-level hydraulic system is given, as well as a kinematics model and hydraulic system model.

Compared to the traditional OPS, the proposed TPS can provide a high-pressure or low-pressure oil supply based on the load of the hydraulic actuators required to meet the robot’s motion, which reduces throttling loss by enhancing the matching between the system pressure and the actuator’s required pressure. The experimental and simulation results further confirm the energy-saving advantages of the TPS. In the simulation, the average power consumption of the robot with the TPS was 1.079 kW, representing a 39.13% reduction compared to the average 1.77 kW of power required by the OPS. Similarly, in the experiment, the TPS demonstrated a power consumption of 8.3 kW, which is a 28.8% reduction compared to the 11.7 kW of power consumed by the OPS. The significant difference in power consumption between the experimental and simulation results can primarily be attributed to the industrial-grade valves used in the experiment, which have considerable leakage loss. This loss was not accounted for in the simulation. From the results, it can be seen that the TPS exhibits pressure fluctuations during pressure switching, which may affect its control accuracy. However, the reduction in accuracy is minimal and within acceptable limits. Therefore, given its superior energy-saving performance, the TPS is well suited for use in heavy-load robots. Future work will focus on optimizing its mechanical structure, integrating hydraulic components, and reducing weight to minimize energy loss and enhance control accuracy during robot movement.

## Figures and Tables

**Table 1:** Information on the main sensors used in the measurement and control system.

Sensor Type	Sensor Model	Measurement Range	Accuracy
IMU	LPMS-RS232AL2	Roll: ±180°; Pitch: ±90°; Yaw: ±180°;	<0.5°; 2° RMS
Angle encoder	R22H 0505 W270EM	0–270°	0.3% FS
Pressure sensor	MIK-P300	0–25 MPa	0.5% FS
Force sensor	CHHBM-1	0–1 t; 0–1.5 t	0.1% FS
Force sensor	BSLM-3	0–2000 kg	0.1% FS
Current sensor	HSTS016L	200 A	1% FS

**Table 2:** Constraints on the foot trajectory during its swing phase.

	X Direction	Z Direction
Position t=0	$S/4+s_{i}$	$-H_{a}$
Position t=T/4	$s_{i}$	$-H_{a}+h$
Position t=T/2	$-S/4+s_{i}$	$-H_{a}$
Velocity t=0	−S/T	0
Velocity t=T/2	−S/T	0
Acceleration t=0	0	0
Acceleration t=T/2	0	0

**Table 3:** Hydraulic system parameters.

Parameters	Value	Unit
$V_{pl1}$	$2.81\times 10^{-5}$	$m^{3}$
$V_{pl2}$	$5.18\times 10^{-5}$	$m^{3}$
$A_{1}$	0.0013	$m^{2}$
$A_{2}$	$7.6576\times 10^{-4}$	$m^{2}$
$\beta_{e}$	$7\times 10^{8}$	${\mathrm{N/m}}^{2}$
$k_{q}$	$3.5635\times 10^{-7}$	$m^{3}$Pa/s
$\omega_{v}$	314.16	rad/s
ζ	0.707	

**Table 4:** Ground contact parameters.

Parameter	Value	Unit
Stiffness	$2.855\times 10^{6}$	N/m
Force exponent	2.2	
Damping	$1\times 10^{6}$	N/(m/s)
Penetration depth	$1\times 10^{-4}$	m
Static coefficient	0.7	
Dynamic coefficient	0.55	
Stiction transition velocity	0.1	m/s
Friction transition velocity	10	m/s

## Data Availability

All the data presented in this study are available within the main text.

## References

[1] Suzumori K., Faudzi A.A. (2018). Trends in hydraulic actuators and components in legged and tough robots: A review. Adv. Robot..

[2] He J., Gao F. (2020). Mechanism, actuation, perception, and control of highly dynamic multilegged robots: A review. Chin. J. Mech. Eng..

[3] Raibert M., Blankespoor K., Nelson G., Playter R. (2008). Bigdog, the rough-terrain quadruped robot. IFAC Proc. Vol..

[4] Semini C., Tsagarakis N.G., Guglielmino E., Focchi M., Cannella F., Caldwell D.G. (2011). Design of HyQ—A hydraulically and electrically actuated quadruped robot. Proc. Inst. Mech. Eng. Part I J. Syst. Control Eng..

[5] Semini C., Barasuol V., Goldsmith J., Frigerio M., Focchi M., Gao Y., Caldwell D.G. (2016). Design of the hydraulically actuated, torque-controlled quadruped robot HyQ2Max. IEEE/ASME Trans. Mechatron..

[6] Khan H., Kitano S., Frigerio M., Camurri M., Barasuol V., Featherstone R., Caldwell D.G., Semini C. Development of the lightweight hydraulic quadruped robot—MiniHyQ. Proceedings of the 2015 IEEE International Conference on Technologies for Practical Robot Applications (TePRA).

[7] Yang K., Zhou L., Rong X., Li Y. (2018). Onboard hydraulic system controller design for quadruped robot driven by gasoline engine. Mechatronics.

[8] Chai H., Meng J., Rong X., Li Y. (2014). Design and implementation of scalf, an advanced hydraulic quadruped robot. Robot.

[9] Irawan A., Nonami K., Ohroku H., Akutsu Y., Imamura S. (2011). Adaptive impedance control with compliant body balance for hydraulically driven hexapod robot. J. Syst. Des. Dyn..

[10] Nonami K., Huang Q., Komizo D., Fukao Y., Asai Y., Shiraishi Y., Fujimoto M., Ikedo Y. (2003). Development and control of mine detection robot COMET-II and COMET-III. JSME Int. J. Ser. Mech. Syst. Mach. Elem. Manuf..

[11] Davliakos I., Roditis I., Lika K., Breki C.M., Papadopoulos E. (2018). Design, development, and control of a tough electrohydraulic hexapod robot for subsea operations. Adv. Robot..

[12] Hu N., Li S., Huang D., Gao F. (2015). Gait planning and control of quadruped robot with high payload. J. Syst. Simul..

[13] Cho J., Kim J.T., Park S., Lee Y., Kim K. JINPOONG, posture control for the external force. Proceedings of the IEEE ISR 2013.

[14] Tong Z., Ye Z., Gao H., He J., Deng Z. Electro-hydraulic control system and frequency analysis for a hydraulically driven six-legged robot. Proceedings of the 2016 IEEE International Conference on Aircraft Utility Systems (AUS).

[15] Li M., Jiang Z., Wang P., Sun L., Ge S.S. (2014). Control of a quadruped robot with bionic springy legs in trotting gait. J. Bionic Eng..

[16] Seok S., Wang A., Chuah M.Y., Hyun D.J., Lee J., Otten D.M., Lang J.H., Kim S. (2014). Design principles for energy-efficient legged locomotion and implementation on the MIT cheetah robot. IEEE/ASME Trans. Mechatron..

[17] Yang K., Rong X., Zhou L., Li Y. (2019). Modeling and analysis on energy consumption of hydraulic quadruped robot for optimal trot motion control. Appl. Sci..

[18] Sun Y., Hua Z., Li Y., Hui C., Li X., Su B. (2021). Modeling and analysis on low energy consumption foot trajectory for hydraulic actuated quadruped robot. Int. J. Adv. Robot. Syst..

[19] Tani K., Nabae H., Hirota Y., Endo G., Suzumori K. Walking trajectory design of hydraulic legged robot with limited powered pump. Proceedings of the 2021 IEEE International Conference on Robotics and Automation (ICRA).

[20] Zhai S., Jin B., Cheng Y. (2020). Mechanical design and gait optimization of hydraulic hexapod robot based on energy conservation. Appl. Sci..

[21] Liu X., Rossi A., Poulakakis I. (2018). A switchable parallel elastic actuator and its application to leg design for running robots. IEEE/ASME Trans. Mechatron..

[22] Yin X., Yan J., Wen S., Zhang J., Pang M. (2023). Origami-Based Decoupling Clutch Achieves Energy-Efficient Legged Robots. IEEE Robot. Autom. Lett..

[23] Mazumdar A., Spencer S., Salton J., Hobart C., Love J., Dullea K., Kuehl M., Blada T., Quigley M., Smith J. Using parallel stiffness to achieve improved locomotive efficiency with the Sandia STEPPR robot. Proceedings of the 2015 IEEE International Conference on Robotics and Automation (ICRA).

[24] Mazumdar A., Spencer S.J., Hobart C., Salton J., Quigley M., Wu T., Bertrand S., Pratt J., Buerger S.P. (2017). Parallel Elastic Elements Improve Energy Efficiency on the STEPPR Bipedal Walking Robot. IEEE/ASME Trans. Mechatron..

[25] Fan W., Dai Z., Zhang B., He L., Pan M., Yi J., Liu T. (2024). HyExo: A Novel Quasi-Passive Hydraulic Exoskeleton for Load-Carrying Augmentation. IEEE/ASME Trans. Mechatron..

[26] Fan W., Dai Z., Li W., Liu T. (2024). Load-Carrying Assistance of Articulated Legged Robots Based on Hydrostatic Support. IEEE Robot. Autom. Lett..

[27] Xue Y., Yang J., Shang J., Wang Z. (2014). Energy efficient fluid power in autonomous legged robotics based on bionic multi-stage energy supply. Adv. Robot..

[28] Hua Z., Zhang Z., Chai X., Sun Y. (2023). Energy efficiency onboard hydraulic power for quadruped robot based on high-low double pumps supply. J. Mech. Sci. Technol..

[29] Xue Y., Yang J., Shang J., Xie H. (2015). Design and optimization of a new kind of hydraulic cylinder for mobile robots. Proc. Inst. Mech. Eng. Part C J. Mech. Eng. Sci..

[30] Hua Z., Rong X., Li Y., Chai H., Li B., Zhang S. (2020). Analysis and Verification on Energy Consumption of the Quadruped Robot with Passive Compliant Hydraulic Servo Actuator. Appl. Sci..

[31] Liu Z., Jin B., Dong J., Zhai S., Tang X. (2022). Design and Control of a Hydraulic Hexapod Robot with a Two-Stage Supply Pressure Hydraulic System. Machines.

[32] Junlong W. (2017). Research on Energy Saving Strategy of Quadruped Robot Hydraulic System. Master’s Thesis.

